# Supplementary material for: Dynamic disorganization of synaptic NMDA receptors triggered by autoantibodies from psychotic patients
Source: Nat Commun. 2017 Nov 27;8:1791. doi: 10.1038/s41467-017-01700-3 (PMC5702610; doi:10.1038/s41467-017-01700-3)
Supplement: Supplementary file 1 — Supplementary Information [file 41467_2017_1700_MOESM1_ESM.pdf]

Supplementary Figure 1

Jezequel et al.

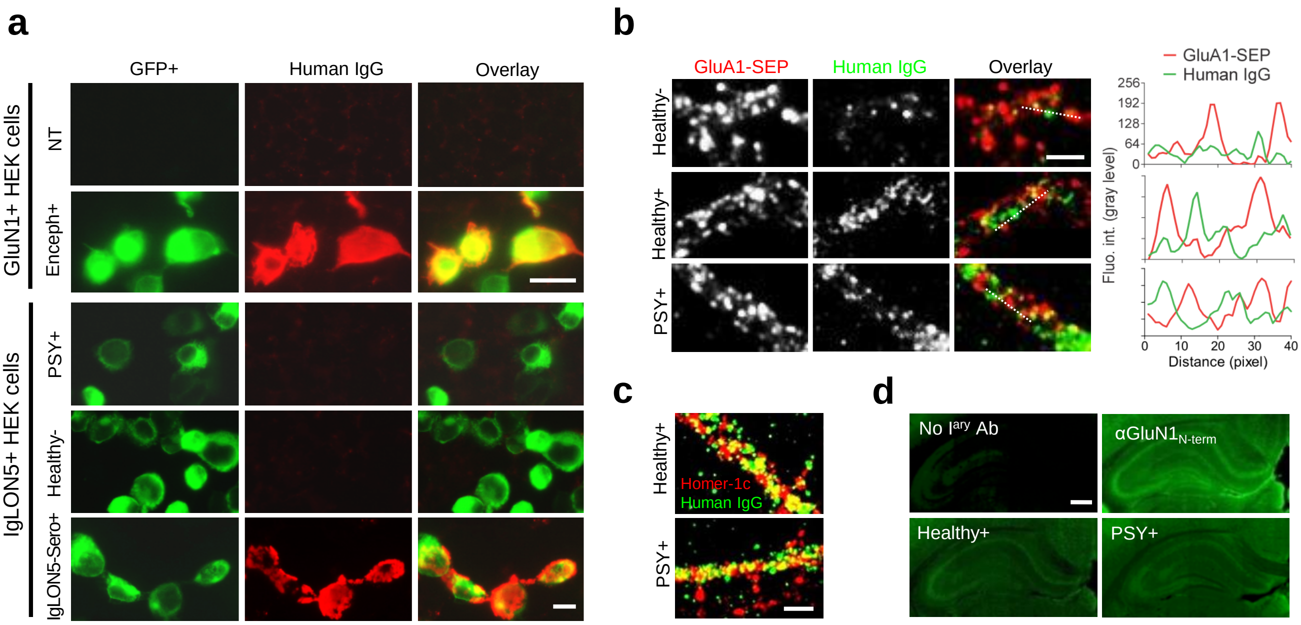

Supplementary Figure 1. Characterization of circulating autoantibodies' target

(**a**) *Upper panel:* Control immunostaining using non-transfected or HEK293 cells expressing GluN1-GFP and GluN2B subunits exposed to the sera of a patient with NMDAR-Ab encephalitis (1/10, 3h incubation). Note the complete overlap between serum reactivity (red) and GFP-positive HEK cells (green) in presence of Enceph+ serum. Scale bar, 10 µm. *Lower panel:* Control immunostaining using IgLON5-GFP+ HEK293 cells exposed to the sera of PSY+, Healthy- or IgLON5+ subjects. Note the absence of staining in presence of serum from PSY+ and Healthy- samples. Scale bar, 10 µm. (**b**) Surface co-immunostaining for GluA1-SEP containing AMPAR (red) and human IgG's target (5µg/ml, green) in live hippocampal neurons (11 div). Scale bar, 2µm. *Right panel*: corresponding linescans plotting GluA1-SEP and human IgG fluorescence intensities over distance (pixel). No overlap in any of the conditions was observed. (**c**) Representative dendritic areas of cultured hippocampal neurons labelled with purified IgG (5 µg/ml, green) from Healthy+ or PSY+ samples and Homer-1c (red) which localizes glutamate postsynaptic densities. Both staining reveal a cluster-type distribution with good co-localization with the synaptic area. Scale bar, 20 µm. (**d**) Immunostaining of mice hippocampal slices (P22) incubated with a commercial $\alpha GluN1_{N\text{-}term}$ antibody (20 µg/ml, 3h) or purified IgG from Healthy+ (20 µg/ml), PSY+ (20 µg/ml) or an anti-human Alexa 488 without any primary antibody (no primary Ab). Note the similar staining pattern between all conditions in presence of primary antibodies. Scale bar, 50 µm.

Supplementary Figure 2

Jezequel et al.

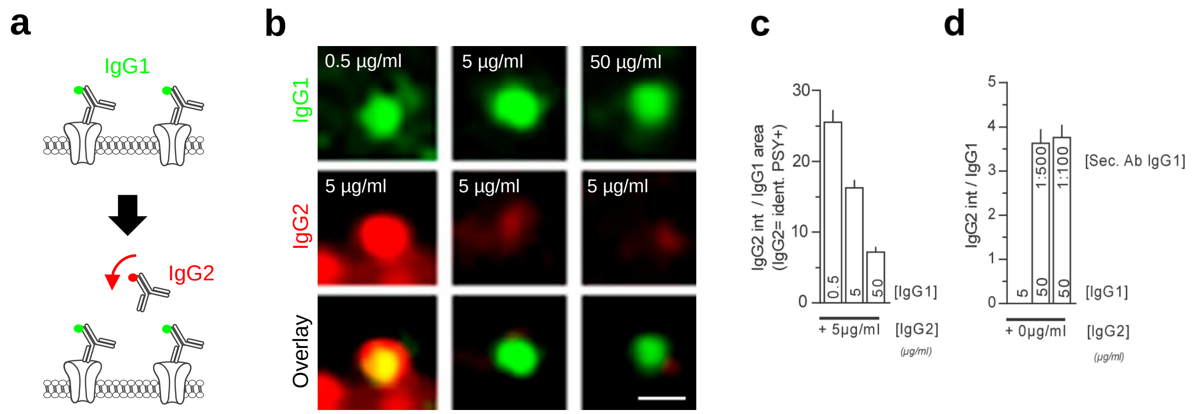

Supplementary Figure 2. Optimization of immuno-competition test. (**a**) Experimental design of the immuno-competition assay. Hippocampal neurons were first incubated with PSY+ NMDAR-Ab (IgG1) labeled in green, followed by a secondary exposure (IgG2, red). The IgG2 was either identical or different from IgG1. (**b**) Representative fluorescence staining after incubation with a PSY+ NMDAR-Ab (IgG1, green) at different concentrations (0.5, 5, or 50 µg/ml, overnight, 4°C) followed by incubation with the same IgG (IgG2 red, 5 µg/ml, overnight, 4°C). Scale bar, 400 nm. Note that IgG1 were first coupled with a secondary anti-human Alexa 488 and then remaining antigen binding sites were blocked using anti-human Fab fragments to reduce unspecific labelling. The decrease in IgG2 staining indicates the lack of binding sites for IgG2. (**c**) Quantification of mean fluorescence intensity of IgG2 within IgG1 cluster areas and comparison using different concentrations of IgG1: 0.5 µg/ml (n = 25 cluster fields, N = 14 neurons), 5 µg/ml (n = 30, N = 16) and 50 µg/ml (n = 33, N = 14). (**d**) A high concentration of IgG1 produces unspecific red staining. The red staining (no IgG2 with 1:500 or 1:100 secondary IgG2) was measured and compared after 5 and 50 µg/ml IgG1 concentrations. Data are expressed as mean fluorescence intensity ± SEM (5µg/ml IgG1: n = 14 cluster fields, N = 8 neurons; 50 µg/ml IgG1 / 1:500: n = 20, N = 8; 50 µg/ml IgG1 / 1:100: n = 19, N = 8). Note that 50 µg/ml IgG1 produce an unspecific red staining, irrespective of the IgG2 secondary antibody dilution. The IgG1 concentration of 5µg/ml was then selected as the best concentration for immuno-competition experiments.

Supplementary Figure 3

Jezequel et al.

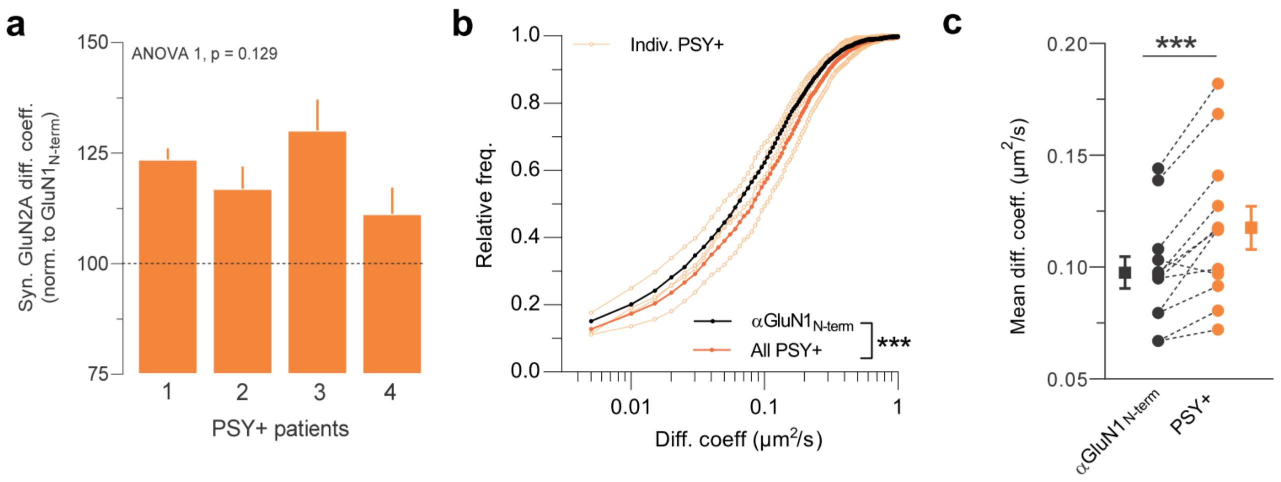

Supplementary Figure 3. Intra-variability within the PSY+ group. (**a**) NMDAR-Ab from different PSY+ patients comparably affect synaptic GluN2A-NMDAR instantaneous diffusion coefficient. Normalized GluN2A-NMDAR diffusion coefficient (normalization to the control condition αGluN1$_{N\text{-}term}$) obtained with purified IgG from PSY+ patient 1 (124 ± 2.5%, n = 2641 trajectories, N = 22 neurons), PSY+ patient 2 (117 ± 5.0%, n = 928, N = 9), PSY+ patient 3 (130 ± 6.9%, n = 517, N = 9) or PSY+ patient 4 (111 ± 5.9%, n = 369, N = 19). p = 0.129, one-way ANOVA. (**b**) Cumulative distributions of instantaneous diffusion coefficient of synaptic GluN2A-NMDAR exposed to αGluN1$_{N\text{-}term}$ antibody (n = 3975 trajectories, N = 66 neurons) or PSY+ NMDAR-Ab (all PSY+ patients, n = 2959, N = 59). 3 out of 4 PSY+ NMDAR-Ab induced a right shift in the distribution when compared to the control group (***p < 0.001, Kolmogorov-Smirnov test). (**c**). Comparison of mean GluN2A diffusion coefficient values after exposure for 30 min to a control αGluN1$_{N\text{-}term}$ antibody (0.098 ± 0.007 µm^2/s, n = 12 experiments) or PSY+ NMDAR-Ab (0.118 ± 0.010 µm^2/s, n = 12). Each plotted dot-pair corresponds to the mean diffusion coefficient value of an independent experiment. Each patient and experiment with PSY+ NMDAR-Ab increased the instantaneous diffusion coefficient of synaptic GluN2A-NMDAR compared to the control condition (*** p < 0.001 two-tailed paired Student *t*-test).

Supplementary Figure 4

Jezequel et al.

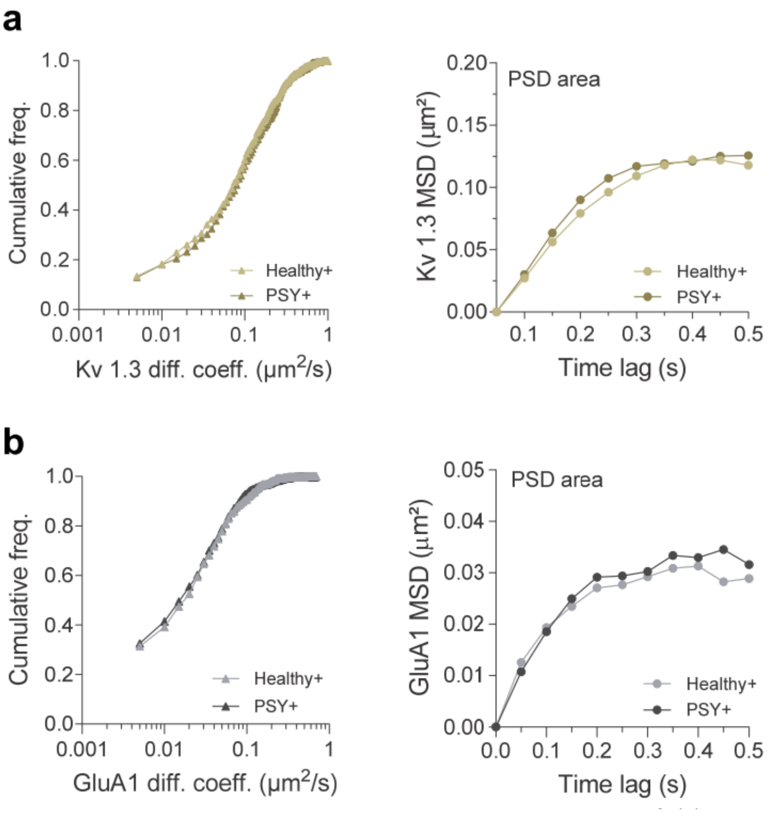

Supplementary Figure 4. NMDAR-Ab from PSY+ patients and healthy subjects do not alter potassium channel and AMPA receptor dynamics in synapses. (**a**) Cumulative distributions of the instantaneous diffusion coefficient of synaptic Kv1.3 (Median diffusion coefficient ± 25-75% IQR, Healthy+ = 0.075 $\mu m^2/s$, IQR= 0.021-0.166 $\mu m^2/s$, n = 580 trajectories, N = 6 neurons; PSY+ = 0.084 $\mu m^2/s$, IQR= 0.026-0.190 $\mu m^2/s$, n = 598, N = 7; p = 0.311, Mann-Whitney test) and MSD curves (p = 0.996, Kolmogorov-Smirnov test) in the PSD area. (**b**) Cumulative distributions of the instantaneous diffusion coefficient of synaptic GluA1-AMPAR (Median diffusion coefficient ± 25-75% IQR, Healthy+ = 0.018 $\mu m^2/s$, IQR= 0.037–0.049 $\mu m^2/s$, n = 699 trajectories, N = 10; PSY+ = 0.015 $\mu m^2/s$, IQR= 0.004-0.041 $\mu m^2/s$, n = 1616, N = 14; p = 0.0505, Mann-Whitney test) and MSD curves (p = 0.249, Kolmogorov-Smirnov test) in the PSD area.

Supplementary Figure 5

Jezequel et al.

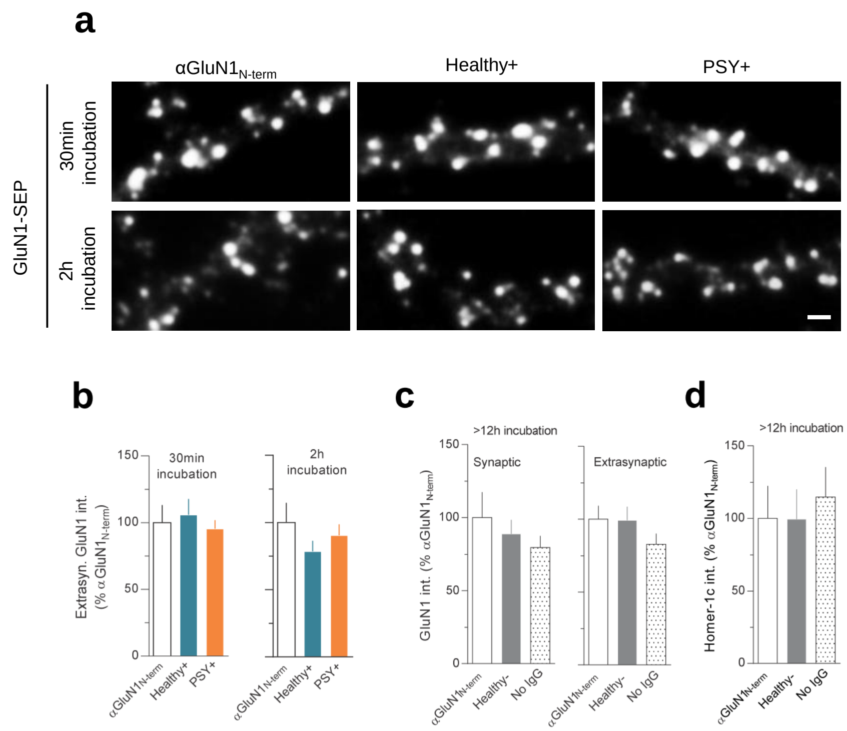

Supplementary Figure 5. Further characterization of NMDAR-Ab and control antibodies' effect on NMDAR surface expression. (**a**) Surface live staining of GluN1-SEP clusters in hippocampal neurons exposed 30min or 2h with either a control antibody ($\alpha GluN1_{N\text{-}term}$) or NMDAR-Ab (Healthy+ or PSY+ NMDAR-Ab). Scale bar, 1 µm. (**b**) Normalized extrasynaptic GluN1-SEP fluorescence intensity after 30min incubation with $\alpha GluN1_{N\text{-}term}$ (100 ± 13%, n = 18 neurons), Healthy+ (106 ± 12%, n = 36) or PSY+ (95 ± 6%, n = 55) NMDAR-Ab ; and after a 2h-exposure to $\alpha GluN1_{N\text{-}term}$ (100 ± 15%, n = 23 neurons), Healthy+ (78.5 ± 8%, n = 48) or PSY+ (91 ± 8%, n = 76) NMDAR-Ab. $p > 0.05$, one-way ANOVA. For each experiment, data were normalized to the respective control condition ($\alpha GluN1_{N\text{-}term}$) and expressed as mean ± SEM. $p > 0.05$, one-way ANOVA. (**c**) Normalized GluN1-SEP fluorescence intensity after a 12h-exposure to $\alpha GluN1_{N\text{-}term}$ (100 ± 8%, n = 17), Healthy- (89 ± 10%, n = 29) or no IgG (80 ± 8%, n = 29) in synaptic areas and after a 12h-exposure to $\alpha GluN1_{N\text{-}term}$ (100 ± 9%, n = 17), Healthy- (99 ± 9%, n = 29) or no IgG (83 ± 7%, n = 29) in extrasynaptic compartments ($p>0.05$, one-way ANOVA). (**d**) Normalized Homer-1c fluorescence intensity after 12h incubation with $\alpha GluN1_{N\text{-}term}$ (100 ± 22%, n = 17 neurons), Healthy- (99 ± 21%, n = 29) or no IgG (115 ± 21%, n = 29). Data were normalized to the respective control condition ($\alpha GluN1_{N\text{-}term}$) and expressed as mean ± SEM. $p > 0.05$, one-way ANOVA.

Supplementary Figure 6

Jezequel et al.

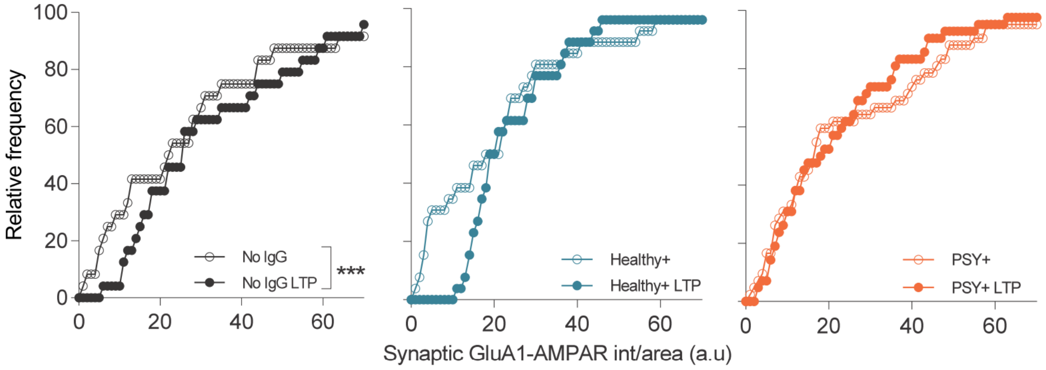

Supplementary Figure 6. Effect of NMDAR-Ab on synaptic GluA1-AMPAR fluorescence intensity after cLTP. Cumulative distributions of synaptic GluA1-SEP AMPAR fluorescence intensity before and after a cLTP stimulus on neurons pre-exposed for 12h to no IgG (n = 24 neurons), Healthy+ (n = 29) or PSY+ NMDAR-Ab (n = 42) subjects. Values of GluA1-SEP fluorescence intensity were normalized to the area of interest. In control condition (no IgG), the cLTP stimulus triggers an increase of synaptic GluA1-SEP intensity as the distribution is shifted to the right (***$p < 0.001$, Kolmogorov-Smirnov test). In neurons exposed to PSY+ NMDAR-Ab, a slight decrease of GluA1-SEP fluorescence intensity is observed after cLTP ($p > 0.05$, Kolmogorov-Smirnov test) whereas a subpopulation of GluA1-SEP clusters still responds to cLTP after exposure to Healthy+ NMDAR-Ab ($p > 0.05$, Kolmogorov-Smirnov test).

Supplementary Table 1

Jezequel et al.

	**Healthy**	Healthy -	Healthy +	**SCZ**	PSY-	PSY+
Number	104	101	3 (2.9%)	48	39	9 (18.7%)<br>(p = 0.01 vs healthy+)
Gender (F)	55.7%	58.4%	100%	21.3%	20.5%	33.3 %
Age	37.42 (13)	36.7 (14.7)	35.6 (3.3)	36.0 (11.5)	36 (12)	34.6 (9.8)
Years of education	13.04 (2.6)			11.39 (2.6)		
Anti-psychotic treatment	NA	NA	NA	35 (74.5%)		
Age at onset of principal disease	NA	NA	NA	24.5 (7.2)		
First episode	NA	NA	NA	10 (24.4%)	12.5%	26.5%
PANSS_tot	NA	NA	NA	68.63 (20)	64.71 (17.4)	84.63 (22.3)
PANSS_pos	NA	NA	NA	15.83 (6.7)	14.6 (5.8)	20.11 (8.2)
PANSS_Neg	NA	NA	NA	18.91 (8.2)	18.29 (8.1)	20.67 (8.8)
PANSS_G	NA	NA	NA	34.07 (9.9)	32.11 (8.6)	43.25 (10.2)
Other Antibodies	ANA 42/101<br>DNA 7/101<br>ANCA 8/101<br>SM 8/101<br>TPO 11/101	ANA 40/101 (39.6%)<br>DNA 6/101 (5.9%)<br>ANCA 8/101 (7.9%)<br>SM 8/101 (7.9%)<br>TPO 11/101 (10.9%)	ANA 2/3 (66.7%)<br>DNA 1/3 (33.3%)<br>ANCA 0/3 (0%)<br>SM 0/3 (0%)<br>TPO 0/3 (0%)	ANA 20/39<br>DNA 9/39<br>ANCA 5/39<br>SM 5/39<br>TPO 2/38	ANA 16/39 (41%)<br>DNA 6/39 (15,4%)<br>ANCA 4/39 (10,2%)<br>SM 2/39 (5,1%)<br>TPO 1/38 (2,6%)	ANA 4/9 (44.4%)<br>DNA 3/9 (33.3%)<br>ANCA 1/9 (11.1%)<br>SM 3/9 (33.3%)<br>TPO 1/9 (11.1%)
Infectious screening	HSV1 67/101<br>CMV 69/101<br>Toxo 53/101	HSV1 65/101 (64.3%)<br>CMV 67/101 (66.3%)<br>Toxo 53/101 (52.5%)	HSV1 2/3 (66.7%)<br>CMV 2/3 (66.7%)<br>Toxo 0/3 (0%)	HSV1 26/39<br>CMV 27/39<br>Toxo 33/39	HSV1 22/39 (56.4%)<br>CMV 20/39 (51.3%)<br>Toxo 27/39 (69.2%)	HSV1 4/9 (44.4%)<br>CMV 7/9 (77.8%)<br>Toxo 6/9 (66.7%)

Supplementary Table 1. Demographic and clinical features of the studied population. PANSS_tot = Positive and Negative Syndrome Scale Total Score; PANNS_pos = Positive and Negative Syndrome Scale Positive Score; PANSS_Neg = Positive and Negative Syndrome Scale Negative Score; PANSS_G = Positive and Negative Syndrome Scale General Score. ANA = anti-nuclear; ANCA = anti-neutrophilic cytoplasmic; CMV = Cytomegalovirus; DNA = anti double-stranded DNA; HSV1 = Herpes simplex virus 1; SM = anti-smooth muscle; TPO = anti-thyroid peroxidase; Toxo = Toxoplasmosis

Supplementary Table 2

Jezequel et al.

	PSY+ (n = 9)	*PSY- (n = 39)*	*P value*
	n (%)		
Sex (% men)	6 (67%)	31 (79%)	*0.773*
Acute state (% acute)	7 (78%)	24 (61.5%)	*0.305*
Cannabis (% positive)	2 (22%)	7 (18%)	*0.593*
First Episode	1	9	*0.659*
Season of birth	1	13	*0.453*
	Mean (SD)		
Age	36 (12)	35.6 (12)	*0.978*
BMI	28 (9.9)	24.28 (5.1)	*0.599*
Number of episodes	5.0 (5.6)	4.76 (4.7)	*0.938*
Age at onset of principal dis	24.4 (6.9)	24.52 (7.3)	*0.951*
Panss_tot	87.71 (22.2)	64.71 (17.4)	*0.01*
Panss_pos	21.25 (8)	14.6 (5.8)	*0.026*
Panss_Neg	21.63 (8.9)	18.29 (8.1)	*0.332*
Panss_G	43.85 (10.9)	32.11 (8.6)	*0.0075*
Duration of illness	11.62 (8.2)	11.08 (9.4)	*0.737*

Supplementary Table 2. Comparative demographic and clinical features of seropositive versus seronegative schizophrenia patients. BMI = Body Mass Index; PANSS_tot = Positive and Negative Syndrome Scale Total Score; PANNS_pos = Positive and Negative Syndrome Scale Positive Score; PANSS_Neg = Positive and Negative Syndrome Scale Negative Score; PANSS_G = Positive and Negative Syndrome Scale General Score.

Supplementary Table 3

Jezequel et al.

Pts	Age/ Gender	Age at onset	PANSS total	Current Treatment	Residual / active symptoms	CSF	EEG	Brain MRI	Other medical history
1	36/M	18	79	Clozapine 500mg/d<br>Valproic acid 2000mg/d<br>Biperidene 4mg/d	Dissociation<br>Cognitive impairment	< 10 cells/mm3<br>Prot 636 mg/l<br>Elevated Alb ratio	Normal	Nle	None
2	44/M	22	66	Risperidone 2mg/d<br>Cyamemazine 25mg/d<br>Diazepam 5mg/d	Persistant dissociation<br>Cognitive impairment	< 10 cells/mm3<br>Prot 328 mg/l<br>Nle	Left temporo-occipital theta slow waves with few spike-waves	Nle	Diabetes mellitus Type 2
3	28/M	25	132	Clozapine 500mg/d<br>Levomepromazine 50mg/d<br>Tropatepine 10mg/d	Negative symptoms: blunted affect<br>Cognitive impairment	< 10 cells/mm3<br>Prot 543mg/l<br>Elevated Alb ratio	Bi-frontal theta slow waves with few spike-waves	Nle	None
4	47/F	30	99	Aripiprazole 10mg/d<br>Tropatepine 10mg/d	Persistantcoenesthetic hallucination, persecutive delusion<br>Dissociation<br>Cognitive impairment	< 10 cells/mm3<br>Prot 520mg/l<br>OCB +	Normal	Nle	Multiple ovarian cysts
5	21/F	16	63	Clozapine 350 mg/d<br>Tropatepine 10mg/d<br>Propanolol 40 mg/d	Negative symptoms: blunted affect<br>Cognitive impairment	< 10 cells/mm3<br>Prot 718mg/l<br>Elevated Alb ratio	diffuse sporadic theta slow waves	Nle	Multinodulargoiter<br>Ovarian Sertoli-Leydig tumour<br>Willebrand disease type 1<br>Multiple ovarian cysts
6	31/M	17	73	NA	NA	NA	NA	NA	NA
7	46/F	38	64	NA	NA	NA	NA	NA	NA
8	24/M	21	87	NA	NA	NA	NA	NA	NA
9	27/M	24	78	NA	NA	NA	NA	NA	NA

ElevatedAlb ratio = Elevated Albumine CSF/serum ratio; OCB = oligoclonal bands; Nle = Normal

Supplementary Table 3. Characteristics of schizophrenia patients with serum NMDAR-Ab at the second time of assessment. Elevated Alb ratio = Elevated Albumine CSF/serum ratio; OCB = oligoclonal bands; Nle = Normal.

Supplementary Table 4

Jezequel et al.

Figure	Parameter	Conditions	Values<br>Mean ± SEM or Median ± 25%-75% IQR	N	Statistical test<br>α= 0.05	P value
**2d**	IgG2 int / IgG1 area ratio	PSY+/ident. PSY+<br>PSY+/diff. PSY+<br>PSY+/Healthy+<br>PSY+/Enceph.+	5.551 ± 0.351<br>8.629 ± 0.804<br>10.345 ± 0.999<br>10.437 ± 1.078	38 (10 cells / exp)<br>99 (32 cells / exp)<br>87 (26 cells / exp)<br>51 (15 cells / exp)	Kolmogorov-Smirnov test	PSY+/ident. PSY+ vs PSY+/diff. PSY+= 0.0453, p*<br>PSY+/ident. PSY+ vs PSY+/Healthy+= 0.0123, p*<br>PSY+/ident. PSY+ vs PSY+/Enceph.+= 0.0088, p**
**3c**	Synaptic GluN2A instantaneous diffusion coefficient	Healthy-<br>αGluN1$_{N\text{-}term}$<br>Healthy+<br>PSY+	0.009 μm²/s, IQR = 0.025 - 0.050 μm²/s<br>0.056 μm²/s, IQR = 0.015 - 0.139 μm²/s<br>0.055 μm²/s, IQR = 0.014 - 0.136 μm²/s<br>0.076 μm²/s, IQR= 0.020 - 0.177 μm²/s	94 (13 cells/4 exp)<br>749 (52 cells/13 exp)<br>313 (42 cells/5 exp)<br>695 (59 cells/10 exp)	Kruskal-Wallis followed by Dunn's multiple comparison test	Kruskal-Wallis statistic= 38.41, p<0.0001<br><br>*Dunn's (mean rank difference detail)*<br>αGluN1$_{N\text{-}term}$ vs Healthy-= 247.4, p***<br>αGluN1$_{N\text{-}term}$ vs Healthy+= 17.49, ns<br>αGluN1$_{N\text{-}term}$ vs PSY+= - 91.12, p**<br>Healthy- vs Healthy+= - 229.9, p**<br>Healthy- vs PSY+= - 338.5, p***<br>Healthy+ vs PSY+= -108.6, p*
**3e**	Exchange frequency	Healthy+<br>PSY+	2.31 ± 0.06 Hz<br>2.68 ± 0.07 Hz	241 (42 cells/5 exp)<br>268 (59 cells/10 exp)	Two-tailed Mann-Whitney test	Mann-Whitney U= 25815<br>*** p<0.0001
**3f**	Perisynaptic GluN2A instantaneous diffusion coefficient	Healthy+<br>PSY+	0.079 μm²/s, IQR = 0.025 - 0.168 μm²/s<br>0.086 μm²/s, IQR = 0.026 - 0.184 μm²/s	1344 (42 cells/5 exp)<br>2264 (59 cells/10 exp)	Two-tailed Mann-Whitney test	Mann-Whitney U= $1{,}484.10^{6}$<br>p= 0.2132, ns
**3g**	Synaptic GluN2A instantaneous diffusion coefficient	Healthy+ 50ng/ml<br>Healthy+ 5μg/ml<br>PSY+ 50ng/ml<br>PSY+ 5μg/ml	0.040 μm²/s, IQR= 0.0135 - 0.091 μm²/s<br>0.030 μm²/s, IQR= 0 - 0.085 μm²/s<br>0.026 μm²/s, IQR = 0.0025 - 0.087 μm²/s<br>0.058 μm²/s, IQR= 0.011 - 0.159 μm²/s	321 (21 cells/5 exp)<br>69 (32 cells/4 exp)<br>187 (24 cells/5 exp)<br>178 (32 cells/4 exp)	Kruskal-Wallis followed by Dunn's multiple comparison test	Kruskal-Wallis statistic= 19.72, ***p= 0.0002<br><br>*Dunn's (mean rank difference detail)*<br>Healthy+ 5μg/ml vs Healthy+ 50ng/ml= - 49.76, ns<br>Healthy+ 5μg/ml vs PSY+ 5μg/ml= -94.34, p*<br>Healthy+ 5μg/ml vs PSY+ 50ng/ml= - 1.269, ns<br>Healthy+ 50ng/ml vs PSY+ 5μg/ml= - 44.58, ns<br>Healthy+ 50ng/ml vs PSY+ 50ng/ml= 48.49, ns<br>PSY+ 5μg/ml vs PSY+ 50ng/ml= 93.07, p***
**3h**	Normalized instantaneous diffusion coefficient	Control<br>PSY+<br>NT-HEK<br>GluN1-HEK	100 ± 3.5%<br>114 ± 2.5%<br>115 ± 2%<br>103 ± 1.6%	1388 (37 cells/3 exp)<br>2658 (59 cells/3 exp)<br>3867 (27 cells/3 exp)<br>4799 (28 cells/3 exp)	Kruskal-Wallis followed by Dunn's multiple comparison test	Kruskal-Wallis statistic= 28.63, ***p<0.0001<br><br>*Dunn's (mean rank difference detail)*<br>GluN1-HEK vs NT-HEK= - 239.6, p*<br>GluN1-HEK vs Control= 301.7, p*

Supplementary Table 4

Jezequel et al.

						GluN1-HEK vs PSY+= - 262.1, ns<br>NT-HEK vs Control= 541.3, p***<br>NT-HEK vs PSY+= 37.58, ns<br>Control vs PSY+= - 503.8, p***
**4c**	GluN2A-NMDAR nano-object numbers	Healthy+<br>PSY+	3.6 ± 0.2<br>4.1 ± 0.2	109 (4 cells/2 exp)<br>112 (5 cells/2 exp)	Two-tailed Mann-Whitney test	Mann-Whitney U= 5267<br>p= 0.0751, ns
**4d**	GluN2A-NMDAR nano-object shape factor	Healthy+<br>PSY+	0.67 ± 0.01 a.u.<br>0.68 ± 0.01 a.u.	368 (4 cells/2 exp)<br>417 (5 cells/2 exp)	Two-tailed Mann-Whitney test	Mann-Whitney U= 73686<br>p= 0.3374, ns
**4e**	GluN2A nano-object area	Healthy+<br>PSY+	13.1 ± 0.47 pixels<br>10.7 ± 0.37 pixels	392 (4 cells/2 exp)<br>458 (5 cells/2 exp)	Two-tailed Mann-Whitney test	Mann-Whitney U= 74499<br>*** p<0.0001
**4h**	Synaptic GluN2A-NMDAR nano-objects number<br><br>Synaptic GluN2A-NMDAR nano-objects area	Healthy+<br>PSY+<br><br><br>Healthy+<br>PSY+	4.3 ± 0.28<br>4.9 ± 0.28<br><br><br>12.8 pixels ± 0.52<br>10.5 pixels ± 0.45	70 (4 cells/2 exp)<br>66 (5 cells/2 exp)<br><br><br>301 (4 cells/2 exp)<br>322 (5 cells/2 exp)	Two-tailed Mann-Whitney test<br><br><br>Two-tailed Mann-Whitney test	Mann-Whitney U= 1928<br>p= 0.0937<br><br><br>Mann-Whitney U= 39747<br>*** p<0.0001
**5b**	Normalized synaptic GluN1-NMDAR fluorescence intensity 30min incubation<br><br>2h incubation<br><br><br><br>>12h incubation	αGluN1$_{N\text{-}term}$<br>Healthy+<br>PSY+<br><br><br>αGluN1$_{N\text{-}term}$<br>Healthy+<br>PSY+<br><br>αGluN1$_{N\text{-}term}$<br>Healthy+<br>PSY+	100 ± 14%<br>88 ± 12.5%<br>80 ± 7%<br><br><br>100 ± 13%<br>136 ± 12%<br>135 ± 12%<br><br>100 ± 14%<br>89.5 ± 12%<br>56 ± 8%	19 (3 exp)<br>36 (3 exp)<br>55 (3 exp)<br><br><br>24 (3 exp)<br>47 (3 exp)<br>77 (3 exp)<br><br>22 (3 exp)<br>34 (3 exp)<br>28 (3 exp)	One-way ANOVA<br><br><br><br><br>One-way ANOVA<br><br><br><br>One-way ANOVA followed by Tukey's multiple comparison test	F value= 0.7604<br>p= 0.4700, ns<br><br><br><br>F value= 1.389<br>p= 0.2527, ns<br><br><br><br>F value= 4.754, *p= 0.0112<br><br>*Tukey's (mean difference detail)*<br>Healthy+ vs PSY+= 19.55, p*<br>Healthy+ vs αGluN1$_{N\text{-}term}$= - 5.839, ns<br>PSY+ vs αGluN1$_{N\text{-}term}$= - 25.39, p*

Supplementary Table 4

Jezequel et al.

**5c**	Normalized extrasynaptic GluN1-NMDAR fluorescence intensity >12h incubation	αGluN1$_{N\text{-}term}$<br>Healthy+<br>PSY+	84 ± 12%<br>92 ± 3%<br>91 ± 4%	14 (3 exp)<br>34 (3 exp)<br>30 (3 exp)	Kruskal-Wallis test	Kruskal-Wallis statistic= 2.869<br>p= 0 .2382, ns
**5d**	Normalized Homer-1c fluorescence intensity >12h incubation	αGluN1$_{N\text{-}term}$<br>Healthy+<br>PSY+	238 ± 56%<br>226 ± 38%<br>185.5 ± 41%	25 (3 exp)<br>31 (3 exp)<br>16 (3 exp)	One-way ANOVA	F value= 0.1073<br>p= 0.8984, ns
**6b**	Synaptic EphB2R clusters area	Healthy-<br>Healthy+<br>PSY+	0.041 ± 0.013 μm^2<br>0.046 ± 0.017 μm^2<br>0.037 ± 0.012 μm^2	24 (16 cells/2 exp)<br>49 (7 cells/2 exp)<br>40 (5 cells /2 exp)	Kruskal-Wallis test followed by Dunn's multiple comparison test	Kruskal-Wallis statistic= 6.652, *p= 0.0359<br><br>*Dunn's (mean rank difference detail)*<br>Healthy- vs Healthy+= - 6.588, ns<br>Healthy- vs PSY+= 11.37, ns<br>Healthy+ vs PSY+= 17.96, p*
**6c**	Fraction of EphB2R-positive synapses	Healthy-<br>Healthy+<br>PSY+	0.37 ± 0.155<br>0.44 ± 0.19<br>0.315 ± 0.17	25 (16 cells/2 exp)<br>49 (7 cells/2 exp)<br>41 (5 cells/2 exp)	Kruskal-Wallis test followed by Dunn's multiple comparison test	Kruskal-Wallis statistic= 14.04, ***p= 0.0009<br><br>*Dunn's (mean rank difference detail)*<br>Healthy- vs Healthy+= - 11.97, ns<br>Healthy- vs PSY+= 14.47, ns<br>Healthy+ vs PSY+= 26.45, p***
**6e**	Synaptic EphB2R instantaneous diffusion coefficient (cumulative distribution)	Healthy+<br>PSY+	0.128 $\mu m^2/s$, IQR = 0.043 - 0.260 $\mu m^2/s$<br>0.154 $\mu m^2/s$, IQR = 0.058 - 0.302 $\mu m^2/s$	1432 (33 cells/3 exp)<br>1104 (39 cells/3 exp)	Kolmogorov-Smirnov test	Kolmogorov-Smirnov D= 0.1391<br>**p= 0.0025
**7b**	Normalized frequency of spontaneous NMDAR-mediated Ca^{2+} transients	No IgG<br>Healthy-<br>Healthy+<br>PSY+	0.52 ± 0.07<br>0.60 ± 0.05<br>0.52 ± 0.06<br>0.55 ± 0.04	38 (4 cells/10 exp)<br>38 (5 cells/10 exp)<br>68 (10 cells/10 exp)<br>131 (15 cells/10 exp)	Kruskal-Wallis test followed by Dunn's multiple comparison test	Kruskal-Wallis statistic= 3.551, p= 0.3142<br><br>*Dunn's (mean rank difference detail)*<br>Healthy+ vs PSY+= - 10.67, ns<br>Healthy+ vs Healthy-= - 29.96, ns<br>Healthy+ vs no IgG= - 6.673, ns<br>PSY+ vs Healthy-= - 19.29, ns<br>PSY+ vs no IgG= 4.000, ns<br>Healthy- vs no IgG= 23.29, ns

Supplementary Table 4

Jezequel et al.

**8a**	Synaptic GluA1-AMPAR fluorescence intensity	No IgG<br>Healthy-<br>Healthy+<br>PSY+	20.01 ± 13.54 a.u.<br>18.00 ± 8.872 a.u.<br>16.53 ± 10.67 a.u.<br>13.99 ± 9.841 a.u.	74 (24 cells/4 exp)<br>41 (10 cells/2 exp)<br>52 (16 cells/4 exp)<br>74 (20 cells/4 exp)	Kruskal-Wallis test followed by Dunn's multiple comparison test	Kruskal-Wallis statistic = 13.21, ** p= 0.0042<br><br>*Dunn's (mean rank difference detail)*<br>Control vs Healthy-= 1.393, ns<br>Control vs Healthy+= 19.67, ns<br>Control vs PSY+= 38.00, p**<br>Healthy- vs Healthy+= 18.28, ns<br>Healthy- vs PSY+= 36.61, p*<br>Healthy+ vs PSY+= 18.33, ns
**8b**	Normalized synaptic GluA1-AMPAR clusters area	No IgG<br>Healthy+<br>PSY+	120.7 ± 53.25%<br>112 ± 38.39%<br>89.03 ± 23.11%	25 (11 cells/3 exp)<br>26 (10 cells/3 exp)<br>46 (19 cells/3 exp)	One-way ANOVA followed by Tukey's multiple comparison test	F value= 6.873, *** p<0.0001<br><br>*Tukey's (mean difference detail)*<br>No IgG vs Healthy+= 8.708, ns<br>No IgG vs PSY+= 31.64, p**<br>Healthy+ vs PSY+= 22.93, p*
**8f**	Normalized EPSC amplitudes	Healthy-<br>Healthy+<br>PSY+<br>D-AP5	188.5 ± 15.2%<br>163 ± 4.2<br>61 ± 2.9%<br>85 ± 3.1%	8 (4 exp)<br>10 (5 exp)<br>9 (4 exp)<br>5 (4 exp)	One-way ANOVA followed by Tukey's multiple comparison test	F value= 53.74, **p= 0.0016<br><br>*Tukey's (mean difference detail)*<br>Baseline vs Healthy-= - 88.52, p***<br>Baseline vs Healthy+= - 62.62, p***<br>Baseline vs PSY+= 39.16, p**<br>Baseline vs D-AP5= 15.40, ns<br>Healthy- vs Healthy+= 25.90, ns<br>Healthy- vs PSY+= 127.7, p***<br>Healthy- vs D-AP5= 103.9, p***<br>Healthy+ vs PSY+= 101.8, p***<br>Healthy+ vs D-AP5= 78.02, p***<br>PSY+ vs D-AP5= - 23.76, ns
**Suppl.3a**	Normalized GluN2A-NMDAR instantaneous diffusion coefficient	PSY+ 1 patient<br>PSY+ 2 patient<br>PSY+ 3 patient<br>PSY+ 4 patient	124 ± 2.5%<br>117 ± 5.0%<br>130 ± 6.9%<br>111 ± 5.9%	2641 (22 cells/4 exp)<br>928 (9 cells/2 exp)<br>517 (9 cells/2 exp)<br>369 (19 cells/4 exp)	One-way ANOVA	F value= 1.887<br>p= 0 .1294, ns
**Suppl.3b**	Synaptic GluN2A-NMDAR diffusion coefficient (cumulative distributions)	αGluN1$_{N\text{-term}}$<br>All PSY+	0.999 $\mu m^2/s$, IQR = 0.989 - 1.000 $\mu m^2/s$<br>1.000 $\mu m^2/s$, IQR = 0.987 - 1.000 $\mu m^2/s$	3975 (66 cells/13 exp)<br>2959 (59 cells/10 exp)	Kolmogorov-Smirnov test	Kolmogorov-Smirnov D= 0.0746<br>*** p<0.0001
**Suppl.3c**	Mean GluN2A diffusion coefficient	αGluN1$_{N\text{-term}}$<br>All PSY+	0.098 ± 0.007 $\mu m^2/s$<br>0.118 ± 0.010 $\mu m^2/s$	12 exp	Two-tailed paired Student t-test	t= 4.812 df= 11<br>*** p= 0.005

Supplementary Table 4

Jezequel et al.

**Suppl.4a**	Synaptic Kv1.3 instantaneous diffusion coefficient<br><br>Synaptic Kv1.3 MSD	Healthy+<br>PSY+	0.075 µm^2/s, IQR= 0.021 - 0.166 µm^2/s<br>0.084 µm^2/s, IQR= 0.026 - 0.190 µm^2/s	580 (12 cells/2 exp)<br>598 (7 cells/2 exp)	Two-tailed Mann-Whitney test<br><br>Kolmogorov-Smirnov test	Mann-Whitney U= 167510<br>p= 0.3114, ns<br><br>Kolmogorov-Smirnov D= 0.1667<br>p= 0.9963, ns
**Suppl.4b**	Synaptic GluA1-AMPAR instantaneous diffusion coefficient<br><br>Synaptic GluA1-AMPAR MSD	Healthy+<br>PSY+	0.018 µm^2/s, IQR= 0.037 - 0.049 µm^2/s<br>0.015 µm^2/s, IQR= 0.004 -0.041 µm^2/s	699 (10 cells/4 exp)<br>1616 (14 cells/4 exp)	Two-tailed Mann-Whitney test<br><br>Kolmogorov-Smirnov test	Mann-Whitney U= 8607<br>p= 0.1140, ns<br><br>Kolmogorov-Smirnov D= 0.4167<br>p= 0.2485, ns
**Suppl.5b**	Normalized extrasynaptic GluN1-SEP fluorescence intensity<br>30min incubation<br><br>2h incubation	$\alpha GluN1_{N-term}$<br>Healthy+<br>PSY+<br><br>$\alpha GluN1_{N-term}$<br>Healthy+<br>PSY+	100 ± 13%<br>106 ± 12%<br>95 ± 6%<br><br>100 ± 15%<br>78.5 ± 8%<br>91 ± 8%	18 (3 exp)<br>36 (3 exp)<br>55 (3exp)<br><br>23 (3 exp)<br>48 (3 exp)<br>76 (3 exp)	One-way ANOVA<br><br>One-way ANOVA	F value= 0.3504<br>p= 0.7052, ns<br><br>F value= 0.39224<br>p= 0.3999, ns
**Suppl.5c**	Normalized synaptic GluN1-SEP fluorescence intensity > 12h incubation<br><br>Normalized extrasynaptic GluN1-SEP fluorescence intensity > 12h incubation	$\alpha GluN1_{N-term}$<br>Healthy-<br>No IgG<br><br>$\alpha GluN1_{N-term}$<br>Healthy-<br>No IgG	100 ± 8%<br>89 ± 10%<br>80 ± 8%<br><br>100 ± 9%<br>99 ± 9%<br>83 ± 7%	17 (3 exp)<br>29 (3 exp)<br>29 (3 exp)<br><br>Same data set	One-way ANOVA<br><br>One-way ANOVA	F value= 0.7758<br>p= 0.4641, ns<br><br>F value= 1.307<br>p= 0.2771, ns
**Suppl.5d**	Normalized Homer-1c fluorescence intensity<br>> 12h incubation	$\alpha GluN1_{N-term}$<br>Healthy-<br>No IgG	100 ± 22%<br>99 ± 21%<br>115 ± 21%	17 (3 exp)<br>29 (3 exp)<br>29 (3 exp)	One-way ANOVA	F value= 0.1757<br>p= 0.8392, ns
**Suppl. 6**	Synaptic GluA1-SEP AMPAR fluorescence intensity (cumulative distributions)	No IgG<br>No IgG LTP<br><br>Healthy+<br>Healthy+ LTP<br><br>PSY+<br>PSY+ LTP	27.01 ± 4.68 a.u.<br>33.32 ± 4.94 a.u.<br><br>23.27 ± 4.87 a.u.<br>27.25 ± 4.22 a.u.<br><br>24.76 ± 3.24 a.u.<br>23.24 ± 2.83 a.u.	24 pairs (4 exp)<br><br>26 pairs (4 exp)<br><br>42 pairs (4 exp)	Kolmogorov-Smirnov test<br><br>Kolmogorov-Smirnov test<br><br>Kolmogorov-Smirnov test	Kolmogorov-Smirnov D= 0.2764<br>*** p= 0.0002<br><br>Kolmogorov-Smirnov D= 0.1057<br>p= 0.4980, ns<br><br>Kolmogorov-Smirnov D= 0.1138<br>p= 0.4030, ns

Supplementary Table 4. Statistical analysis details

Supplementary Methods

Participants. Patients (both in- and outpatients) with schizophrenia (n = 48) meeting the DSM-IV criteria (APA, 1994), consecutively consulted/admitted to two university-affiliated psychiatric departments, (Mondor Hospital, Créteil, University of Paris-Est and Fernand Widal Hospital, Paris, University of Diderot, France) were included in the present study after approval by a French ethical committee and written informed consent from the participants. Healthy subjects (n= 104) were enrolled through a clinical investigation center (Center for Biological Resources, Mondor Hospital, Créteil, France). The control group was matched with the schizophrenic sample for age, gender and years of education. Only individuals without a personal or first degree family history of psychotic disorders, affective disorders, addictive or suicidal behavior, as measured by the Family Interview for Genetic Studies (FIGS) and also without a personal or family history of autoimmune diseases (information obtained either from controls/patients or from the first degree relatives or from medical records) were included. Other exclusion criteria were: i) current or past immunosuppressive treatment, ii) recent infection or ongoing inflammatory disease *viz* arthritis, ankylosing spondylitis, Crohn's disease, asthma, systemic lupus erythematous, iii) a positive serology for HIV1/2; Hepatitis A, B and C prior to enrollment, and iv) neurological disorder with cognitive impairment *viz* multiple sclerosis, Parkinson's disease, head injury, cerebrovascular accident, Alzheimer's disease. Five patients (two females and three males) were re-hospitalized for thorough clinical investigation (Supplementary Table 3).

Clinical evaluation. Patients were interviewed with the French version of the "Diagnostic Interview for Genetic Studies" (DIGS) for the assessment of lifetime clinical characteristics of schizophrenia as well as for demographic characteristics (*i.e.* number of years of education, working status, season of birth). Current medications as well as hospitalization status were recorded. Manic symptoms were assessed with the Young Mania Rating Scale (YMRS) and depressive symptoms with the Montgomery and Asberg Depression Rating Scale (MADRS) for bipolar disorder and the Calgary Depression Scale for schizophrenia (CDS). Positive and negative symptoms were assessed using the Positive and Negative Syndrome Scale (PANSS). Current smoking status using the Fagerström scale and recent or past alcohol or drug abuse were recorded for all the participants.

Cognitive evaluation. All participants were evaluated for episodic verbal memory using the California Verbal Learning Test (CVLT). Working memory (backward digit span and letter number sequencing) was evaluated using the Wechsler Adult Intelligence Scale III (WAIS). Premorbid IQ was assessed with the National Adult Reading Test (NART), which estimates premorbid ability level from a word reading test which provides an estimate of vocabulary size.

Encephalitis patients. Sera and purified immunoglobulins were collected in anti-NMDAR encephalitis patients at symptom presentation, before any treatment and stored at -80°C in NeuroBioTec (Biobank of the Hospices Civils de Lyon, France). As previously reported, the presence of GluN1 antibodies was demonstrated, and all samples were dialyzed against phosphate buffered saline, and solutions were used at pH of 7.4 (see [1]).

Collection of blood samples and medical exam. Blood samples were collected from patient and control groups within one week of the clinical assessment. Sera were purified in order to extract IgG isotype antibodies. Samples were then dialyzed against phosphate-buffered saline and solutions were used at pH 7,4. Subjects who were seropositive for NMDAR-Ab were contacted for additional medical and biological evaluations. These patients underwent: i) a lumbar puncture to identify the presence of NMDAR-Ab in the cerebrospinal fluid (CSF), measure cell counts, CSF proteins, glucose, and CSF/serum albumin ratio, which is an indicator of blood-brain barrier impairment; ii) a tumor screening (females had a pelvic MRI and males had a thoracic-abdominal-pelvic CT scan (TAP CT scan)); iii) an additional blood test to measure and confirm the presence of NMDAR-Ab as well as antibodies to neurotropic pathogens, such as Toxoplasma Gondii (*T.gondii*), herpes simplex virus I (HSV I) and cytomegalovirus (CMV), and to measure cytokine concentrations; and iv) an electroencephalograph (EEG) to identify spikes indicative of seizures or other abnormalities.

Infectious antibody analysis. The reactivity of immunoglobulin G (IgG) and immunoglobulin M (IgM) classes antibodies to *T.gondii*, HSV I and CMV were measured using solid phase-enzyme microplate immunoassay methods (IBL America, USA). The results were quantified by calculating the ratio of the reactivity of the samples to a standard sample run on each microplate. *T. gondii* IgG seropositivity was defined as a *T. gondii*/IgG ratio ≥ 0.8, equivalent to ≥ 10 international units. The Stanley Laboratory of Developmental Neurovirology (USA) conducted blindly the antibody measurements (Supplementary Table 3).

Cell-based assay. Serum samples were tested for the presence of NMDAR-Ab using a cell-based assay on human embryonic kidney cells (HEK293) ectopically expressing both GluN1-GFP (positioned at the C-terminus) and GluN2B-NMDAR subunits, as previously described[1,2]. Briefly, HEK293 were grown on glass coverslips in Dulbecco's modified Eagle's medium (DMEM) with 10% fetal calf serum (FCS). 24h later, cells were co-transfected (Lipofectamine LTX, Invitrogen) with plasmids coding for GluN1 and GluN2B subunits of the NMDAR. To visualize transfected cells, GluN1 was fused to a GFP (Green Fluorescent Protein). Cells were grown in the presence of NMDAR antagonists (500 µM ketamine) for 42h and then incubated for 1h in a saturation buffer (DMEM, 25mM HEPES, 1% BSA, 5% normal goat serum). Live cells were incubated with human's serum (1/10 in saturation buffer) as a primary antibody for 3h at room temperature, subsequently washed in DMEM-HEPES and fixed in 1% paraformaldehyde (PFA) for 15min. Cells were eventually incubated with a Cy3-conjugated anti-human antibody for 1h. Bound antibodies were visualized using an epifluorescence microscope (Axiophot, Zeiss). The observed labeling was scored from 0 to 4 by three independent blinded observers, the final score representing the median of the scores from 3 independent assays. Samples were considered as positive when the final score exceeded a threshold of 2-3.

Immuno-absorption. HEK293 were prepared as described above (see "Cell-based assay" paragraph). 24h after plating (150 000 cells/ml), cells were transfected with GluN1-SEP and GluN2B (Lipofectamine LTX, Invitrogen). After 4h incubation with the DNA mix, medium was replaced by fresh, equilibrated and heated supplemented DMEM medium. The following day, either transfected or non-transfected HEK cells were incubated for 1h with

NMDAR-Ab (5 µg/ml). This step was repeated 6 times and the resulting absorbed fraction was kept at 4°C (maximum 24h) for further live experiments.

Primary cell culture and protein expression. Cultures of hippocampal neurons were prepared from E18 Sprague-Dawley rats. Cells were plated at a density of 50 x 10^3 cells per ml on poly-lysine pre-coated coverslips. Coverslips were maintained in a 3% horse serum containing Neurobasal medium (Invitrogen). After a few days *in vitro* (*div*), the original plating medium was replaced by a serum-free medium. Cultures were maintained at 37°C in 5% CO_2 for 15 *div* at maximum. For exogenous protein expression, 7-10 *div* hippocampal cultured neurons were transfected at least 48h before each experiment using either the Effectene (Qiagen) or phosphate calcium transfection[3].

Immunochemistry

Immunohistochemistry. The pattern staining obtained with human IgG was assessed using immunohistochemistry on hippocampal slices. For this, 22 postnatal day-old mice were perfused with 4% PFA. Coronal sections of 50 µm were obtained on a vibratome (Leica) and incubated overnight at 4°C with either a polyclonal antibody against the N-terminal part of the GluN1 subunit ($\alpha GluN1_{N\text{-}term}$ Alomone Labs, 20 µg/ml) or purified IgG from healthy (Healthy+, 20 µg/ml) and psychotic (PSY+, 20 µg/ml) subjects. Fluorescent revelation was carried out with secondary anti-rabbit or anti-human Alexa 488 antibodies (Life Technologies, 1/1000) for 2h at room temperature. Images were obtained using a Nanozoomer and a confocal microscope (SP8, Leica).

In another series, brains (provided by A. Ramsey) from wild-type and GluN1-KD animals at 14 weeks were perfused and stored at -20°C. Coronal tissue sections of hippocampal areas (20 µm thick) were cut on a microtome-cryostat, thaw-mounted onto Thermo Scientific, SuperFrost Ultra Plus adhesion slides, and stored at -20°C until further processing. Sections were fixed at 4°C in 4% paraformaldehyde. Blocking was carried out in a 1xTBS solution in 0.3M glycine containing 10% normal goat serum (Sigma) and incubation with PSY+ purified IgG (5 µg/ml, pooled from 2 different patients) was done in a 1xTBS solution containing 10% normal goat serum overnight at 4°C. Staining with secondary antibody anti-human Alexa 568 (Invitrogen, 1/500) was performed for 1h and slides were mounted with Vectashield + Dapi (Vector Laboratories). Image acquisition was done on a video spinning-disk system (Leica DMI6000B, 40X).

Immunocytochemistry. The pattern staining obtained with human IgG was also assessed in dissociated cells. Purified IgG were incubated as primary antibodies (5 µg/ml) on fixed neurons (4% PFA, 15min, 10 *div*). Cells were then carefully washed and incubated with a secondary anti-human Alexa 488 antibody (Life Technologies, 1/500, 30min). To assess purified IgG's effect on NMDAR and EphB2R surface content, live neurons were incubated with either commercial or human IgG (30min, 2h or >12h, 37°C). Surface exogenous GluN1-SEP receptors were then specifically stained using a monoclonal antibody against GFP (Roche, 1/500, 20min, 37°C) and Alexa 488-conjugated anti-mouse secondary antibody (Invitrogen, 1/500, 45min). Surface endogenous EphB2R were labeled with a polyclonal antibody against the extracellular part of EphrinB2R (R&D, 1/200, 3h, 37°C) and a secondary anti-goat Alexa 488 antibody (Invitrogen, 1/500, 30min). In order to label the post-synaptic density, neurons were fixed (4% PFA, 15min), permeabilized with Triton-BSA 1% (5min) and

successively incubated with an anti-Homer-1c antibody (Synaptic systems, 1/500, 30min) and a secondary anti-guinea pig Alexa 594 antibody (Jackson ImmunoResearch, 1/500, 30min). All imaging sessions were done on a video spinning-disk system (Leica DMI6000B, 63X) and quantification analysis was performed using MetaMorph software (Molecular Devices) and ImageJ (NIH).

Immunocompetition

Brain tissue. Snap frozen, non-perfused 7 µm thick sagittal sections of Sprague-Dawley rat brains were used for this experiment. Sections of tissue were fixed (4% PFA, 15min), washed in PBS, and incubated with undiluted serum from patients with schizophrenia, anti-NMDAR encephalitis, or blood donors overnight at 4°C. Sections were then extensively washed with cold PBS and incubated for 1h at 4°C with biotinylated IgG from a representative patient with anti-NMDAR antibodies. After washing, the binding of biotinylated IgG was demonstrated with a standard avidin-biotin-peroxidase method (Vectastain ABC kit Elite, PK-6100, Vector). Slides were then mildly counterstained with hematoxylin, mounted, and results photographed with a digital camera (AxioCam MRc) adapted to a confocal microscope (Zeiss LSM710).

Hippocampal neurons. After fixation (4% PFA, 15min), hippocampal cultures (12 *div*) were treated with a blocking solution (PBS-BSA 1%, 30min) and successively incubated with PSY+ NMDAR-Ab (purified IgG 5 µg/ml, overnight, 4°C) and a secondary anti-human Alexa 488 antibody (Invitrogen, 1/500, 1h). Remaining antigen binding sites were blocked using anti-human Fab fragments (Jackson ImmunoResearch, 100 µg/ml, 1h). Cells were then incubated with competing IgG from either PSY+, Healthy+, or Enceph+ individuals (purified IgG 5 µg/ml, overnight, 4°C), and bound antibodies were eventually stained with a secondary anti-human Alexa 568 antibody (Invitrogen, 1/500, 1h). All imaging sessions and quantification analysis were performed as for immunocytochemistry.

Direct stochastic optical reconstruction microscopy (dSTORM). Live neurons were incubated with either Healthy+ or PSY+ NMDAR-Ab for 2h at 37°C. Surface endogenous GluN2A-NMDAR were then specifically stained using an anti-GluN2A antibody (Agrobio, 0.1 mg/ml, 15min). After fixation (4% PFA, 15min), neurons were permeabilized with 0.4% Triton X-100 (5min) and treated with a blocking solution containing 1.5% BSA/0.1% fish gel/0.1% Triton X-100 for 40min. Cells were then successively incubated with an anti-PSD-95 antibody (Thermo Scientific, 1/500, 45min) and secondary anti-rabbit Alexa 647 (Invitrogen, 0.1 mg/ml, 30min) and anti-mouse Alexa 532 (Invitrogen, 0.1 mg/ml, 30min) antibodies. A second fixation was performed after incubation with the secondary antibodies. All imaging sessions were performed using a Leica SR GSD 3D microscope (Leica HC PL APO 160x 1.43 NA oil immersion TIRF objective) and an ANDOR EMCCD iXon camera. Samples were illuminated in TIRF mode and images were obtained with an exposure time of 10.85 ms with up to 100,000 consecutive frames. Imaging was carried out at room temperature in a closed Ludin chamber (Life Imaging Services) using pH-adjusted extracellular solution containing oxygen scavengers and reducing agents. Image acquisition was controlled by the Leica LAS software. An initial high power 642 nm laser was used to convert the fluorescence into the dark state in order to reach a desired density for single molecule detection.

During acquisition, the 642 nm laser power was kept at a constant low level while the 405 nm laser power was adjusted to keep an optimal level of stochastically activated molecules per frame. Localization of single molecules and reconstruction of the super resolved image was performed by applying a fitting algorithm determining the centroid-coordinates of a single molecule and fitting the point-spread-function (PSF) of a distinct diffraction limited event to a Gaussian function. The final achieved spatial resolution was 40 nm. Multicolor fluorescent microbeads (Tetraspeck, Invitrogen) were used as fiduciary markers to correct for lateral drifts. GluN2A subunit or PSD-95 protein clusters were identified on the respective epifluorescence images. Structures with a higher intensity than a respective value were identified as clusters. GluN2A subunit nano-objects area and shape were quantified after segmentation of GluN2A subunit dSTORM images (MetaMorph software, Molecular Devices). Morphological features, such as surface area, length and shape of each segmented structure, were exported to calculate their respective distributions. The dimensions were computed by 2D anisotropic Gaussian fitting, from which the principal and the auxiliary axes were extracted as 2.3σ long and 2.3σ short, respectively. The shape factor was calculated as a ratio between the auxiliary and the principal axes. The epifluorescence image of PSD-95 was superimposed on the GluN2A subunit dSTORM image to identify the synaptic nano-objects.

Quantum dot (QD) tracking and surface diffusion calculation. After 30min of preincubation with human IgGs neurons were incubated (10min, 37°C) with primary antibodies against the desired target (see table below). Neurons were then washed and incubated for 10min with QDs. Non-specific binding was blocked by adding 1% BSA (Vector Laboratories) to the QD solution. Green Mitotracker (Life Technologies, 1/2000) was used as an endogenous synaptic marker. QDs were detected by using a mercury lamp, appropriate excitation/emission filters and an EM-CCD camera (Evolve, Photometrics). Images were obtained with an acquisition time of 50ms with 500 consecutive frames. QDs were followed on randomly selected dendritic regions for up to 20min. Recording sessions were processed with the Metamorph software (Universal Imaging Corp). The instantaneous diffusion coefficient, D, was calculated for each trajectory, from linear fits of the first 4 points of the mean square displacement versus time function using *MSD(t)= <r2>(t)= 4Dt.*

Targeted receptor	Primary Ab characteristics	Final concentration	QD characteristics	Final QD dilution
Human IgG's target	Healthy+, PSY+ human	5 µg/ml	655 rabbit F(ab')2 anti-human IgG	1/20 000
GluN2A subunit	Alomone Labs rabbit	1/500 (1.6 µg/ml)	655 goat F(ab')2 anti-rabbit IgG	1/10 000
EphB2R	R&D systems goat	1/200 (1 µg/ml)	655 rabbit F(ab')2 anti-goat IgG	1/2000
GluA1-SEP	Invitrogen mouse	1/500 (0.4 µg/ml)	655 goat F(ab')2 anti-mouse IgG	1/20 000
Kv1.3	Alomone Labs rabbit	1/200 (4 µg/ml)	655 goat F(ab')2 anti-rabbit IgG	1/10 000

Calcium imaging. Dissociated neurons transfected with $GCaMP_3$ at 9-10 div were transferred into a Tyrode solution containing (in mM): 110 NaCl, 5 KCl, 25 HEPES, 15 D-glucose, 2 $CaCl_2$ and 2 $MgCl_2$ (Sigma Aldrich) at 13-15 div. Fifteen minutes before imaging, cells were transferred to a Mg^{2+}-free Tyrode solution with 5 µM Nifedipine (Tocris), and 5 µM Bicuculline (Tocris). Time-lapse images were acquired at 20 Hz. Three time-lapse movies (3000 frames) were successively recorded: 1) “Pre” (baseline period), 2) “Post” (5min after bath application of purified IgG, 5 µg/ml) and 3) “D-AP5” (5min after bath application, 50 µM). Time-lapse movies were concatenated and realigned in ImageJ (PoorMan3DReg plugin, Michael Liebling). Fluorescence from calcium transients *vs.* time was measured within individual ROIs manually defined by the experimenter (ImageJ). All pixels within each ROI were averaged to give a single value time course associated to the ROI. Mean normalized fluorescence (ΔF/F) was calculated by subtracting each value with the mean of the previous 5s values lower than P_{50} (µ) and dividing the result by µ. Positive calcium transients were identified following a two-step procedure: initially, ΔF/F traces were smoothen by convoluting the raw signal with a 10s squared kernel. True positives (with minimum 1s between transients) were then defined on an automated basis using custom-written MATLAB routines where the threshold was set at 5*SD of D-AP5 average trace.

Chemically induced potentiation (cLTP). Live hippocampal neurons transfected with GluA1-SEP were incubated overnight with human IgG (Healthy+ or PSY+, 5 µg/ml, 37°C). After washing thoroughly, chemically induced long-term potentiation (cLTP) was elicited by a bath co-application of glycine (200 µM) and picrotoxin (5 µM) for 4min[4]. cLTP was always applied after a period of baseline acquisition and the medium was carefully replaced by fresh equilibrated and heated medium after induction. GluA1-SEP fluorescence signal was then recorded every 5min during the 30min following the stimulus. Glutamate synapses were defined using the synaptic protein Homer-1c DsRed. Homer-1c clusters were outlined and GluA1-SEP intensity was measured over time within the synaptic areas. Synaptic GluA1-SEP clusters intensity and area values were normalized to the baseline values. All images were collected on a video confocal spinning-disk system (Leica DMI6000B, 63X) and a CoolSNAP HQ2 camera (Photometrics).

***In vivo* hippocampal injection.** Surgical procedures were conducted in accordance with the guidelines of the University of Bordeaux / CNRS Animal Care and Use Committee. Sprague-Dawley rats (P12-15) were anaesthetized with isoflurane (0.8 L/min, 2.5% isoflurane) and mounted on a Kopf stereotaxic frame to maintain the skull stable and allow stereotaxic delivery of purified IgG to the hippocampus. They received an intraperitoneal injection of buprenorphine (0.05 mg/kg) and a local subcutaneous administration of lurocaine (50 µL) to allow full analgesia during and after the surgical procedure. A constant flux of an isoflurane / air mixture (0.4 L/min, 2.5% isoflurane) was delivered through a surgical mask. 1 µl of purified IgG (5 µg/ml) from Healthy-, Healthy+ or PSY+ individuals were infused into the dorsal hippocampus (coordinates relative to bregma, from AP: -3,5 mm, ML: ±2 mm, DV: -2 mm at P12, to AP: -3.8 mm, ML: ±2 mm, DV: -2.5 mm at P15) using borosilicate micropipettes (GC150F-10, Harvard Apparatus, UK) prepared with a horizontal micropipette puller (P-97, Sutter Instrument, USA). After injection, the micropipette was left *in situ* for a couple of minutes

to allow diffusion and reduce reflux up the needle. The incision was then mechanically sutured and rat pups were allowed to recover on a heating blanket before being placed back with their littermates.

Electrophysiology. 2-3h after intra-hippocampal injections, sagittal brain slices (350 µm-thick) were prepared in an ice-cold sucrose buffer solution containing (in mM): 250 sucrose, 2 KCl, 7 MgCl2, 0.5 CaCl2, 1.15 NaH2PO4, 11 glucose, and 26 NaHCO3 (gassed with 95% O2 / 5% CO2). Slices were then incubated for 30 min at 33°C and subsequently stored at room temperature in an artificial CSF (ACSF) solution containing (in mM): 126 NaCl, 3.5 KCl, 2 CaCl2, 1.3 MgCl2, 1.2 NaH2PO4, 25 NaHCO3, and 12.1 glucose (gassed with 95% O2 / 5% CO2; pH 7.35). Whole-cell voltage clamp recordings of CA1 pyramidal cells were performed using infrared differential interference contrast microscopy under continuous perfusion of heated ACSF (32°C) saturated with 95% O2 / 5% CO2. Electrodes (4–5 MΩ) were prepared from borosilicate pipettes (GC150T-10, Harvard Apparatus, UK) with a horizontal micropipette puller (P-97, Sutter Instrument, USA) and filled with a solution containing (in mM): 120 cesium methanesulfonate, 4 NaCl, 4 MgCl2, 10 HEPES, 0.2 EGTA, 4 Na2ATP, 0.33 Na3GTP, and 5 phosphocreatine adjusted to pH 7.3 with CsOH. EPSCs were evoked at a rate of 0.05 Hz using an ACSF-filled glass micropipette positioned in the stratum radiatum to stimulate Schaffer collaterals. Currents were recorded using a Multiclamp 700B amplifier and a Digidata 1550B interface controlled by Clampex 10.7 (Molecular Devices) at -60 mV in the presence of the $GABA_A$ and $GABA_B$ receptor blockers SR95531 (10 µM) and CGP55845 (5 µM), respectively. Under these conditions and after a stable eEPSC recording had been maintained for 10min, tetanic stimulation (four trains of 100 stimuli at 100 Hz, delivered at 20s interval) of Schaffer collaterals was used to induce LTP. The access resistance was monitored throughout the experiment and data were discarded when it changed by > 20%.

Statistical analysis. Univariate analyses were conducted to determine if there were significant differences between patients with schizophrenia and controls, as well as between seropositive and seronegative schizophrenic patients with respect to demographic, clinical, and biological characteristics. For categorical variables, χ^2 test and Fisher's exact test were used while Student's *t*-test and Mann-Whitney U test were used for continuous measures. Comparisons between groups were performed using parametric statistical tests Student's *t*-test or ANOVA followed by a Tukey's Multiple Comparison Test. Comparisons between groups for instantaneous diffusion coefficients and calcium imaging were performed using Mann-Whitney test or Kruskal-Wallis followed by a Dunn's Multiple Comparison Test as the variables are not following normal distributions. For direct comparison of distributions, Kolmogorov-Smirnov test was used. Significance levels were defined as *$p < 0.05$, **$p < 0.01$, ***$p < 0.001$. Statistical analysis was performed using STATA (StataCorp. 2011. Stata Statistical Software: Release 12. College Station, TX: StataCorp LP) and Prism (GraphPad). All values and statistical comparisons are detailed in Supplementary Table 4.

Supplementary references

1. Mikasova L, De Rossi P, Bouchet D, et al. Disrupted surface cross-talk between NMDA and Ephrin-B2 receptors in anti-NMDA encephalitis. *Brain*. 2012;135(Pt 5):1606-1621. doi:10.1093/brain/aws092.
2. Viaccoz A, Desestret V, Ducray F, et al. Clinical specificities of adult male patients with NMDA receptor antibodies encephalitis. *Neurology*. 2014. doi:10.1212/WNL.0000000000000126.
3. Jiang M, Chen G. High Ca 2 + -phosphate transfection efficiency in low-density neuronal cultures. *Nat Protoc*. 2006;1(2). doi:10.1038/nprot.2006.86.
4. Dupuis JP, Ladépêche L, Seth H, et al. Surface dynamics of GluN2B-NMDA receptors controls plasticity of maturing glutamate synapses. *EMBO J*. 2014;33(8):842-861. doi:10.1002/embj.201386356.
